# Computerized Morphometric Analysis of Eryptosis

**DOI:** 10.3389/fphys.2019.01230

**Published:** 2019-09-24

**Authors:** Sanu Susan Jacob, Keerthana Prasad, Pragna Rao, Asha Kamath, Roopa B Hegde, Prathap M Baby, Raghavendra K Rao

**Affiliations:** ^1^Department of Physiology, Kasturba Medical College-Manipal, Manipal Academy of Higher Education, Manipal, India; ^2^School of Information Sciences, Manipal Academy of Higher Education, Manipal, India; ^3^Department of Biochemistry, Kasturba Medical College-Manipal, Manipal Academy of Higher Education, Manipal, India; ^4^Department of Statistics, Prasanna School of Public Health, Manipal Academy of Higher Education, Manipal, India; ^5^Nitte Mahalinga Adyanthaya Memorial Institute of Technology, NITTE, Karkala, India; ^6^Department of Physiology, Melaka Manipal Medical College (Manipal Campus), Manipal Academy of Higher Education, Karnataka, India

**Keywords:** eryptosis, image analysis, morphometry, cell shrinkage, vesiculations, hematology

## Abstract

Eryptosis is the suicidal destruction-process of erythrocytes, much like apoptosis of nucleated cells, in the course of which the stressed red cell undergoes cell-shrinkage, vesiculation and externalization of membrane phosphatidylserine. Currently, there exist numerous methods to detect eryptosis, both morphometrically and biochemically. This study aimed to design a simple but sensitive, automated computerized approach to instantaneously detect eryptotic red cells and quantify their hallmark morphological characteristics. Red cells from 17 healthy volunteers were exposed to normal Ringer and hyperosmotic stress with sodium chloride, following which morphometric comparisons were conducted from their photomicrographs. The proposed method was found to significantly detect and differentiate normal and eryptotic red cells, based on variations in their structural markers. The receiver operating characteristic curve analysis for each of the markers showed a significant discriminatory accuracy with high sensitivity, specificity and area under the curve values. The software-based technique was then validated with RBCs in malaria. This model, quantifies eryptosis morphometrically in real-time, with minimal manual intervention, providing a new window to explore eryptosis triggered by different stressors and diseases and can find wide application in laboratories of hematology, blood banks and medical research.

## Introduction

Erythrocyte is the simplest of all human cells, but endowed with the pivotal role of carrying oxygen from lungs to tissues for metabolism (Jensen, 2009) A decrease in the number of circulating red blood cells (RBCs), due to defect, injury or disease, hampers the oxygen-carrying capacity of blood, resulting in anemia. Typically, RBCs are removed from circulation by 120 days (Piomelli and Seaman, 1993). However, sometimes, it can undergo a premature programmed apoptotic-like death, termed eryptosis, even though it is devoid of a nucleus and mitochondria (Bosman et al., 2005; Lang K.S. et al., 2005). In such cases, RBCs are cleared off from the bloodstream much earlier than its physiological lifespan, limiting the deleterious consequences of intravascular hemolysis (Kiefer and Snyder, 2000). However, if eryptosis is not accompanied by enhanced erythropoiesis, it could facilitate the genesis of anemia (Föller et al., 2008). There are numerous diseases and conditions that are found to have an association with accelerated eryptosis, some of them being iron deficiency anemia (Kempe et al., 2006), malaria (Föller et al., 2009a), bacterial sepsis (Kempe et al., 2007), sickle cell anemia (Weiss et al., 2012), β-thalassemia (Basu et al., 2010), hemolytic uremic syndrome (Lang et al., 2006), glucose-6-phosphate dehydrogenase deficiency (Lang et al., 2002), diabetes (Fırat et al., 2012) and renal insufficiency (Abed et al., 2014).

Currently, there’s a vast ensemble of tests for detection and evaluation of red cell pathology (Wallerstein, 1987; Kuypers et al., 1996). Among all tests, RBC morphology has been observed to be an essential biosensor in predicting the specific disease or condition with which they are associated (Lang et al., 2012). An “eryptotic” RBC exhibits specific hallmark features in its morphology such as cell shrinkage, membrane vesiculation and blebbing (Crawford et al., 2004; Berg et al., 2001; Bratosin et al., 2001). Precise structural analysis of eryptotic RBCs can be studied by scanning electron microscopy (Visser et al., 2017), atomic force spectroscopy (Pretorius et al., 2013), flow cytometry (Attanasio et al., 2015), and more recently, confocal microscopy (Föller et al., 2009b). All of these “state of the art” imaging tools, which although are most accurate and reliable, are also time-consuming, expensive and demands extensive sample-fixing procedures.

It also requires to be emphasized that RBCs are tremendously sensitive and delicate (Gedde et al., 1997), and in the course of all these procedures, extreme care must be taken in the processing, managing and fixing of the samples, to not induce hemolysis (Heireman et al., 2017). Considering that supportive tests are necessary to diagnose the underlying cause of anemia, which is a frequent public health problem in developing and resource-limited countries, it is significant and desirable to improve the quality and accessibility of medical technologies for disease control (Stoltzfus, 2003). For that matter, in such less-funded medical and research facilities, a better, simpler and cost-effective alternative for cellular studies involving morphometric analysis would be to utilize digital technology (Laurinavicius et al., 2012).

The confirmation test used for experimental detection of eryptosis is by measurement of membrane phosphatidylserine exposure. However, there does not exist a single gold-standard tool to assess and quantify morphometrics of eryptosis, *per se*. The focus of this study was to investigate the potential of a quick, automated and reliable software-based image analysis approach to detect as well as compute the degree of eryptosis in real-time, without employing any chemical fixatives or elaborate sample processing, with minimal requirements of proficient personnel and manual analysis. For this, we exposed RBCs to hyperosmotic shock, an already known eryptotic stressor, *in vitro*, and studied the biophysical changes, such as cell shrinkage, formation of vesiculations and disappearance of the central halo.

To test the applicability of this proof-of-concept research study, we have validated the technique in blood samples of patients with malaria. Anemia is a serious dysfunction in malaria, and studies have established the association of eryptosis to be one cause.

## Materials and Methods

### Ethics Statement

This study was approved (IEC 02/2002) by the Institutional Ethics Committee of Kasturba Medical College, Manipal Academy of Higher Education, Manipal. All experimental procedures were conducted following the Ethical Committee guidelines. Written informed consent was obtained from all volunteers. Informed consent was waived for patients with malarial infection and their left-over blood, drawn for clinical investigations, were collected and used for the experiments.

### Blood Sample Collection and Solution Preparation

4 ml heparinized blood samples were extracted from healthy volunteers (*n* = 17). Anticoagulated blood of patients with malaria (*n* = 17) was collected from the Clinical hematology laboratory. Whole anticoagulated blood was centrifuged and washed three times in phosphate-buffered saline, and the plasma and buffy coat were carefully aspirated out after each of the three washes. 1 μL hematocrit was suspended in 2 mL NR (125 mM NaCl, 5 mM KCl, 1 mM CaCl2, 1 mM MgCl2, 32 mM HEPES, 5 mM Glucose; pH = 7.4) and 2 mL hyperosmotic NaCl (Normal Ringer plus 325 mM NaCl). Experiments were conducted in triplicates to ensure reproducibility. All chemicals were purchased from Sigma-Aldrich, India.

A total of 1,912 RBCs, of which 70% (1,338) of randomly selected RBCs, from the healthy samples, were included in the training set for the generation of algorithms and 30% (574) of the total RBCs were included in the testing set. For validation, a small pool of 598 RBCs, from confirmed patients with malaria were included.

### Image Acquisition

**FIGURE 1:**
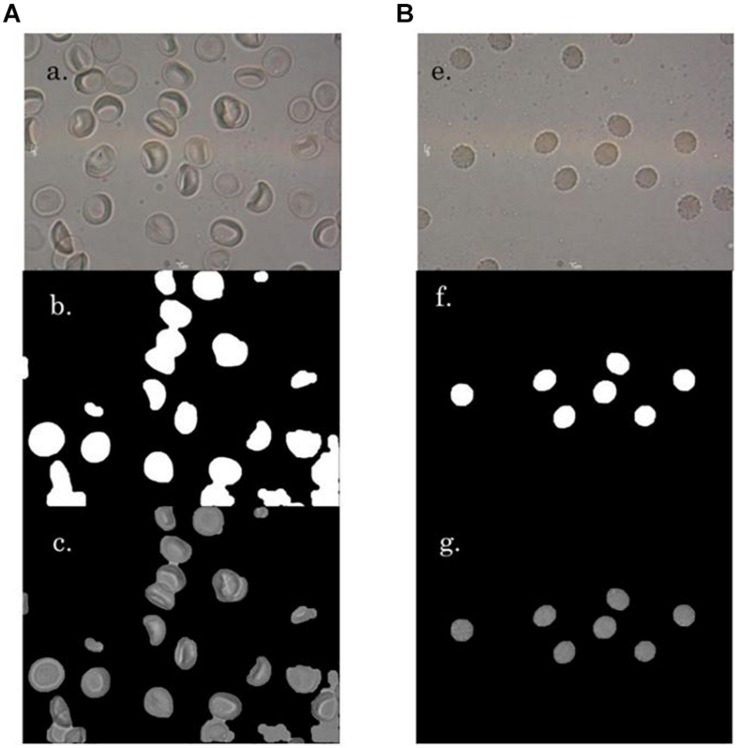
**A** ‘a’ and **B** ‘e’ are representative images of RBCs in NR and hyperosmotic NaCl, respectively. **A** ‘b’ and **B** ‘f’ present the mask generated versions of their respective original images **A** ‘a’ and **B** ‘e’, respectively. **A** ‘c’ and **B** ‘g’ are the background-masked versions, which were generated for detection of RBC regions.

Multiple photomicrographic images of RBCs were captured from diluted blood samples on a glass slide under a 100×oil immersion objective of a compound light microscope mounted with a digital camera (MoticCam 580 5.0MP with software Motic Image Plus 2.0 for PC and Mac), having a display resolution of 1024 × 768 (Figures 1A‘a’,B‘e’). Images of RBCs in hyperosmotic NaCl were captured on immediate exposure of the RBCs with the stressor.

### Computerized Image Analysis

Before running the morphometric analysis, a MATLAB program was written for automatic detection and recognition of RBCs from photomicrographic images. This involved multiple steps of image pre-processing, which enhanced the image for red cell detection, such as, selection of the S component of the HSV (Hue, Saturation and Value) color model representation of the image, contrast enhancement of the S component, followed by thresholding, with a threshold value of 64 operation and morphological closing with disk structuring element of size eight. The small objects and artifacts were eliminated with a morphological area opening step, removing boundary-touching objects as well as objects of sizes less than 3000.

Subsequently, each of the detected RBCs was analyzed for their structural characteristics to determine eryptosis. The “regionprops” function of MATLAB was used for measurements of RBC area, perimeter and the Euler number. The gray level variance within the region of each RBC was also obtained. Based on these features, morphological variations such as cell shrinkage, cell irregularities and the presence of central halos (central pallor) within the RBC were evaluated to categorize the RBC as normal or eryptotic.

#### Cell Shrinkage

**FIGURE 2:**
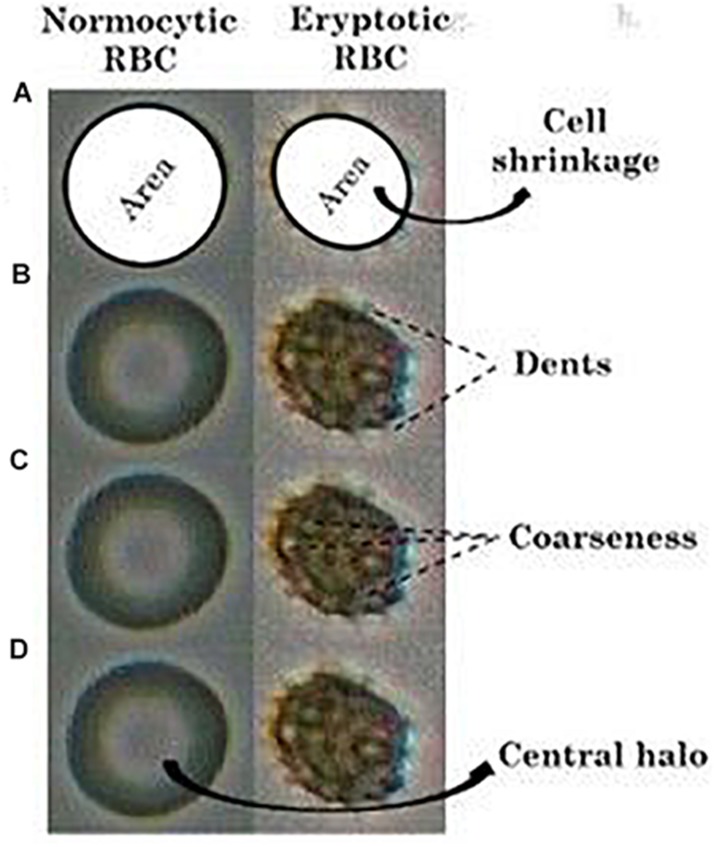
Comparison of **(A)** “area” (shaded), **(B)** “dents,” **(C)** “coarseness” and the **(D)** presence of “central halo” in RBCs in NR and hyperosmotic NaCl solutions. The given images are that of a single normocytic and an eryptotic RBC cropped out and blown up from a larger original image comprising multiple RBCs, after enhancement of their pictorial quality.

For determining red cell size, the red cell regions (red cell area in 2-dimensional view) were picked up, and the number of pixels covered in the region was obtained using the “regionprops” function for “area” in MATLAB. The reduction in the RBC area from the average normal (Figure 2A), was taken as cell-shrinkage. The operational term assigned in this study for cell size was “area.”

#### Cell Irregularities (Membrane Blebbings)

The feature “dents” was used to measure the inflection points along the RBC perimeter, which represents the peripheral cell irregularities (Figure 2B). “Coarseness” measures the gray level variations of the RBC region representing the irregularities within the region (Figure 2C).

##### Computations to extract the feature “dents”

We computed the number of inflection points of RBCs using the FFT approach (Johnson, 2011) to differentiate RBCs in NR and eryptotic RBCs. The steps for calculating the number of inflection points are as follows:

(i) Obtain binary representation *bw* of the original image I
(ii) Obtain boundary coordinates of binary image *bw*(x, y)
(iii) Compute Fast Fourier Transform (FFT) of *x* and *y* coordinates x*_*fft*_* and y*_*fft*_*_,_ respectively
(iv) Compute first-order derivative x*_*d*_* and y*_*d*_* of x*_*fft*_* and y*_*fft*_*_,_ respectively
(v) Compute second-order derivative x*_*dd*_* and y*_*dd*_* of x_*fft*_ and y_*fft*__,_ respectively
(vi) Obtain the real part of first and second-order derivatives d_*x*_, d_*y*_, dd_*x*__,_ and dd_*y*_ of *x* and *y* coordinates respectively
(vii) Compute approximation for closed curvature using equation 1
  *approx* = *sqrt* ((*dd*_*y*_ × *d*_*x*_− *dd*_x^*_)^2^/(*d*_*x*_^2^ + *dy*^2^)^1.5^) (1)
(viii) Check inflection points using the relation *pts* = *find* (*approx* < *t*), where *t* = 0.006
(ix) Determine the difference between adjacent elements of pts

The algorithm results in a vector, which consists of values of the difference between successive values of *pts*. The number of inflections, *N*, is calculated by considering the number of values greater than 1. We get N >/= 2 for eryptotic RBCs and *N* = 1 for normal RBCs.

##### Computations to extract the feature “coarseness”

“Coarseness” represents image granularity. The “coarseness” feature measures the scale of the texture. It gives information on the size of the primitives (basic texture sub-patterns easily identifiable and repeatable, over the entire texture field) making up the texture and has a large value for large primitives or coarse textures (Tegos et al., 2001).

(i) At each pixel *p*(*x, y*), compute six averages for the windows of size *k* = 0, 1, 2,., 5 around the pixel
(ii) At each pixel, compute the absolute differences at each scale *E_*k*_ (x, y)*, between pairs of non-overlapping averages on opposite sides of different directions
(iii) Find the value of *k* that maximizes *E_*k*_ (x, y)* in either direction
(iv) Select the scale with the largest variation: *E*_*k*_ = max *(E_1_, E_2_, E_3_*_,__…_)
(v) The best pixel window size *S*_*best*_ is 2^*k*^
(vi) Compute coarseness by averaging *S*_*best*_ over the entire image

#### Central Halos

When viewed under light microscopy, normal RBCs appear to possess a clear center surrounded by a single halo, owing to its biconcavity. However, once they turn eryptotic, that is, as they shrink and lose their biconcave shape and develop spiculations, this central halo disappears (Figure 2D). To detect the presence or absence of this central halo, we utilized the “Euler number” feature of the “regionprops” function in MATLAB (Gonzalez and Woods, 2008), that measures the topology of the RBC image. In the case of normal RBCs, there exists a single halo (hole) within the RBC (object), and therefore, the Euler number would be computed zero. Under eryptosis, the RBC loses the central halo and develops spiculations (multiple holes), and so the Euler number would be a digit other than zero, which was classified as abnormal.

### Validation of Study

To assess the feasibility of this study, we took the task of testing this method in malaria, following the same steps as described for the healthy group. RBCs from malarial patients (*n* = 17) were exposed to NR and hyperosmotic NaCl. 598 RBCs from malarial samples were included for validation. Photomicrographic images of the RBCs were captured under 100× oil immersion objective, under light microscopy, immediately on suspension. The eryptotic features of the RBCs with operational terms, namely, area, coarseness, dents, and Euler number were analyzed from the photomicrographic images. Comparisons of these features were made between the RBCs of the malarial patients in NR and hyperosmotic shock. Comparisons of these features on RBCs were also made between the healthy and malarial groups.

### Statistical Analysis

The median and inter-quartile range of the eryptotic features between the RBCs in the different groups were reported, and the comparison between the groups was made by Mann Whitney test. To determine the diagnostic accuracy of the tool to quantify the features, receiver-operating characteristic (ROC) curves were generated, in the non-parametric approach, and the cut-off values were defined. Statistical tests were performed using “SPSS” ver. 15.0 (SPSS South Asia, Bangalore) and the statistical significance was defined as a *p*-value ≤ 0.05.

## Results

### Morphological Markers Detected on Normocytic and Eryptotic Rbcs

All the images had been captured without the treatment of the RBCs with any fixatives, and were in their actual live form, in real-time. Normocytic RBCs appeared to be discoid with a central halo and had no membrane vesiculations (Figure 1A). To create a hyperosmotic environment, we added 325 mM sodium chloride (NaCl) on top of NR. As soon as RBCs were exposed to hyperosmotic NaCl, they shrunk due to exosmosis and lost their discoid appearance as well as their central halo (Figure 1B). This phenomenon was accompanied by the formation of membrane vesiculations/spiculations/protrusions/blebs from all around the RBCs. Figure 1 gives a randomly picked representative image of RBCs from each of the two groups that characterized the morphological changes.

### Image Analysis of the Morphological Markers of Eryptosis

After image-processing was done, wherein they were converted to their greyscale version (Figures 1Ab, Bf.) and had their relevant pictorial features extracted, a classification algorithm was developed in MATLAB (version 15a). These programs were generated to analyze precisely four imperative morphological characteristics of eryptotic RBCs, namely its shrinkage, its vesiculations that arose from its perimeter and its center and also the presence or absence of its central halo. Cell shrinkage is a significant hallmark feature of eryptosis, and this was marked by a reduction in its “area” (Figure 2A), computed by the number of pixels covered by the detected RBC in the image. The other striking feature of an eryptotic RBC is that membrane blebs arise from all around it. However, in its two-dimensional image representation, there are those spiculations that were seen to emerge from the periphery, tangentially outward and there were also those that arose from the RBC’s central regions. Features namely “dents” (Figure 2B) and “coarseness” (Figure 2C), representing these cell irregularities from the detected RBCs, were extracted, details of which are elaborated in the “Materials and Methods” section. The disappearance of their central halo, when exposed to hyperosmotic stress as seen in Figure 2D, was computed by the “Euler number” feature, a measure for assessing the topology of a binary image. Figure 2 depicts a representative image of an RBC from each of the two groups, on which the four features have been illustrated.

### Competence of Computerized Image Analysis to Discriminate Normocytic and Eryptotic RBCs

**FIGURE 3:**
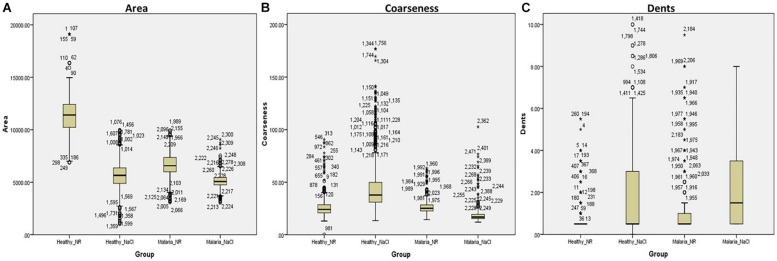
Box-plots showing comparisons of the three features: **(A)** Area, **(B)** Coarseness, and **(C)** Dents, between RBCs of healthy volunteers (*n* = 17) and patients with malaria (*n* = 17) in NR (Healthy_NR, Malaria_NR) and hyperosmotic NaCl solution (Healthy_NaCl, Malaria_NaCl). The box represents the 25th and 75th percentiles and the outliers are represented by open circles and stars.

With the customized programs that were written to detect RBCs and further to compute and quantify the morphological features from them, we obtained clear and significant results for the categorization of normocytic and eryptotic RBCs. The results showed in Table 1 and Figure 3 depicts “area,” “coarseness” and “dents” of RBCs from healthy volunteers in NR and hyperosmotic NaCl, with significant discrimination between the two groups, demonstrating the efficacy of the method for classification of RBCs as normocytic and eryptotic, based on these three features. The presence or absence of the central halo computed by calculating the “Euler number” of the detected RBC also exhibited significant discrimination between the groups as given in Table 3.

**TABLE 1:** Median and inter-quartile ranges of the three features: area, coarseness and dents between RBCs of healthy (*n* = 17) and malaria (*n* = 17) samples in NR and hyperosmotic NaCl solution.

**Markers**	**RBCs from healthy volunteers (Median and IQR)**		**RBCs from malarial samples (Median and IQR)**		***P*-value**
			
	***NR***	***Hyperosmotic NaCl***	***NR***	***Hyperosmotic NaCl***	
Area	11407.0 (10232.0, 12432.75)	5652.0 (4881.50, 6369.75)	6571.0 (5987.75, 7361.75)	5068.0 (4742.0, 5457.0)	<0.0001
Coarseness	24.07 (20.75, 28.55)	37.78 (30.97, 49.89)	25.11 (22.60, 28.51)	16.81 (15.31, 19.44)	<0.0001
Dents	0.5 (0.5, 0.5)	0.5 (0.5, 3.0)	0.5 (0.5,1.0)	1.5 (0.5, 3.5)	<0.0001

*RBCs from healthy volunteers were suspended in NR and hyperosmotic NaCl solutions. Eryptotic RBCs were characterized by significant cell shrinkage (*p* < 0.0001) and cell irregularities: “coarseness” and “dents” (*p* < 0.0001). Validation was performed in RBCs of malarial patients, which produced significant cell shrinkage, coarseness and dents (*p* < 0.0001). *P*-values were determined by Kruskal-Wallis ranked sums and Mann-Whitney test.*

**FIGURE 4:**
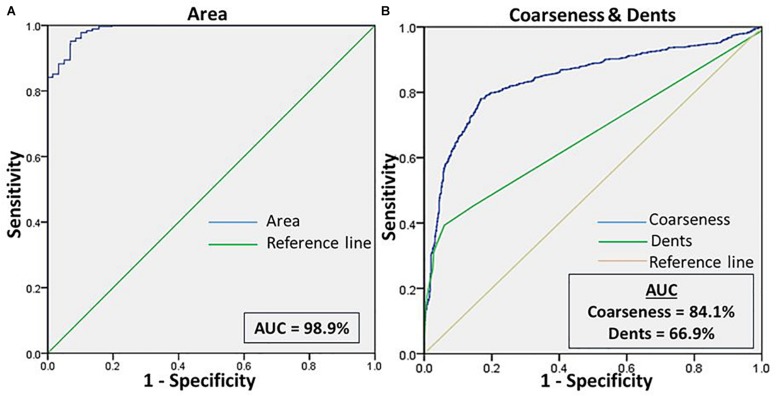
**(A)** Shows the ROC curve for the feature “area” of RBCs, to differentiate the cell size of eryptotic RBCs from normal RBCs. **(B)** Shows the ROC curve analyses for the two features of cell irregularities: “coarseness” and “dents.” This was generated from RBCs of healthy samples in NR and NaCl.

We then assessed the accuracy of this method, to prove them potent discriminatory markers, by generating ROC curves (Figure 4) for each. Table 2 lists the cutoffs, AUC, sensitivity, specificity and the predictive values for the three features between RBCs in different groups. Among the three hallmark structural features, “area” gave the highest sensitivity (95.0%), specificity (93.0%) and AUC of 98.9%, between RBCs of healthy samples in NR and hyperosmotic stress, demarcating cell shrinkage (Figure 4A). Coarseness was next in line, with 80.0% sensitivity, 79.3% specificity and AUC of 84.1% (Figure 4B and Table 2). Figure 4B shows that in terms of “dents” too, there was a significant capability of the algorithm to differentiate normocytic and eryptotic RBCs, depicting a good sensitivity (62.0%), specificity (73.23%) and AUC (66.9%) as displayed in Table 2. The likelihood-ratio (sensitivity/1-specificity) chi-squared tests for the parameters “area,” “oarseness” and “dents” were 13.6, 3.9, and 2.3, respectively, displaying the best for “area.” Table 3 exhibits a 2 × 2 contingency table, portraying measures of association between the true status and the test-predicted status of RBCs classified as normocytic and eryptotic, based on the Euler number feature that computed the presence/absence of the central halo, in NR and hyperosmotic NaCl solutions. The sensitivity, specificity, positive predictive (PPV) and negative predictive (NPV) values obtained for this feature was 98.3, 95.9, 95.7, and 98.3% with an excellent kappa (κ) of 0.94 (0.92–0.96), considering a confidence interval (CI) of 95%.

**TABLE 2:** Test characteristics of the automated tool to classify normocytic and eryptotic RBCs based on the features: area, coarseness and dents, between RBCs of healthy and malaria samples in NR and hyperosmotic NaCl.

**Group**	**Features**	**Cut-off**	**AUC (%)**	**Sensitivity (%)**	**Specificity (%)**	**PPV (%)**	**NPV (%)**	**Likelihood-ratio**
1vs. 2	*Area*	≤8143.0	98.9	95.0	93.0	92.7	95.2	13.6
	*Coarseness*	≥29.42	84.1	80.0	79.3	78.2	81.0	3.9
	*Dents*	≥0.75	66.9	62.0	73.23	74.5	62.6	2.3
1 vs. 3	*Area*	≤8933.0	97.4	96.0	88.1	70.9	98.6	8.1
	*Coarseness*	≥24.56	57.4	56.9	53.3	26.9	80.3	1.2
	*Dents*	≥2.65	56.6	52.7	83.47	58.3	78.5	3.2
1 vs. 4	*Area*	≤8779.50	99.8	99.7	89.8	89.9	99.4	9.8
	*Coarseness*	≥21.54	83.5	80.0	99.4	6.1	54.9	133.3
	*Dents*	≥1.12	76.7	70.0	72.2	56.8	88.4	2.5
3 vs. 4	*Area*	≤7216.5	83.9	81.9	81.2	57.7	93.3	4.4
	*Coarseness*	≥23.09	88.2	86.0	81.3	15.1	16.7	4.6
	*Dents*	≥0.75	69.2	62.9	73.9	70.7	66.6	2.4

*In the given table, parameters depicting cell shrinkage (area) and cell irregularities (“coarseness” and “dents”) exhibited a highly significant sensitivity, specificity, AUC, PPV and likelihood ratio values between normocytic and eryptotic RBCs, between RBCs of healthy samples in NR (Group 1) and NaCl (Group 2). Validation of technique in malaria depicts the capability of the method to detect eryptotic RBCs of malarial samples in NR (Group 3) and NaCl (Group 4). Pair-wise comparisons of the three features between RBCs of all four groups yielded significant test characteristics.*

**TABLE 3:** Test characteristics of the automated tool to classify normocytic and eryptotic RBCs, based on the “Euler number” feature, between RBCs of healthy samples in NR and hyperosmotic NaCl.

	**Test predicted status of RBCs in**	**True status of RBCs**		**Total**
			
		**NaCl solution**	**NR**	
	NaCl solution	906 (98.3%)	41 (4.1%)	947
	NR	16 (1.7%)	949 (72.5%)	965
**Total**		922	990	1912

*Above is a 2 × 2 contingency table showing the probability of classifying the RBCs as normocytic or eryptotic based on the “Euler number” feature. The sensitivity and specificity of the tool were 98.3 and 95.9%, respectively, the positive predictive value (PPV) and negative predictive value (NPV) were calculated to be 95.7 and 98.3%, respectively and the agreement for “Euler number” feature for identifying RBCs as eryptotic or not, using this tool was found to be 0.94 (κ value), with 95% CI (0.92-0.96).*

### Validation of Tool

This method was validated by running the same experiments as described above, on RBCs from patients infected with malaria. Overall, there was significant discrimination (*p* < 0.0001) between RBCs of all groups, analyzed by the Kruskal-Wallis test (Figure 3 and Table 1). There were four groups in all: RBCs of healthy samples in NR and in NaCl and RBCs of malaria samples in NR and in NaCl. Among the four groups, when a pairwise comparison was run between every combination-pairs, there appeared a significant distinction between each pair (*p*-value < 0.001).

**FIGURE 5:**
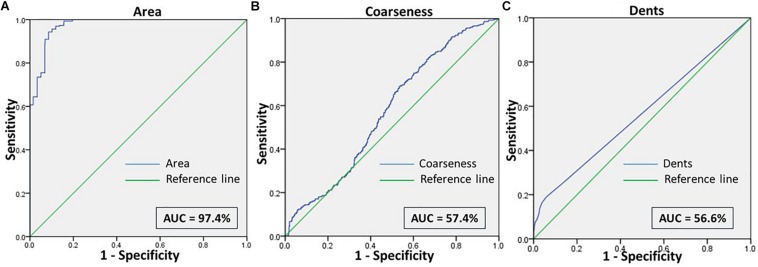
Shows the ROC curve for the features **(A)** Area, **(B)** Coarseness, and **(C)** Dents between RBCs of healthy samples in NR and malaria samples in NR.

When comparing RBCs from healthy and malarial samples in NR, it was observed that, even without hyperosmotic stress, there were apparent differences between RBCs in the two groups, with respect to “area,” “coarseness,” and “dents.” Findings displayed that RBCs in malarial samples showed signs of eryptosis, even before experimental stress (Figure 3 and Tables 1, 5). The sensitivity, specificity and likelihood ratio obtained for the feature “area” were 96.0, 88.1, and 8.07%, respectively, with an AUC of 97.4% (Figure 5 and Table 2). For features “coarseness” and “dents,” the test characteristics obtained were, although lower than that obtained for “area,” very noteworthy. The likelihood ratios acquired for “coarseness” and “dents” were 1.22 and 3.19, respectively (Table 2). The “Euler number” feature also displayed a significant difference between the groups (*p* < 0.001), as presented in the probability distribution Table 5. This establishes the already confirmed fact that RBCs undergo eryptotic characteristics in malaria (Föller et al., 2009a).

When RBCs from malarial samples were stressed hyperosmotically, there was a drastic increase in cell shrinkage, membrane-bleb formation and loss of the central halo (Figure 3 and Tables 1, 4). Among all features, “area” displayed the highest test characteristics between unstressed and hyperosmotically stressed RBCs in malaria. Results obtained for sensitivity, specificity and AUC for “area” were 81.9, 81.2, and 83.9%, respectively, with a satisfactory likelihood ratio of 1.36 (Figure 7 and Table 2). Cell irregularities in terms of “coarseness” exhibited high sensitivity (86.0%) as well as specificity (81.3%), with an AUC of 88.2% and an effective likelihood ratio of 4.53. “Dents” too expressed a good sensitivity (62.9%), specificity (73.9%), AUC (69.2%) and likelihood ratio (2.4). The 2 × 2 contingency table (Table 4), portrays measures of association between true and test-predicted positions of RBCs in these groups, for the “Euler number” feature, exhibiting high sensitivity (83.6%), specificity (82.3%) and a good kappa value of 0.66 with 95% CI (0.60–0.72).

**TABLE 4:** Test characteristics of the automated tool to classify normocytic and eryptotic RBCs, based on the “Euler number” feature, in malarial samples in NR and hyperosmotic NaCl.

	**Test predicted status of RBCs in malaria**	**True status of RBCs in malaria**		**Total**
			
		**NaCl solution**	**NR**	
	NaCl solution	250 (83.6%)	53 (17.7%)	303
	NR	49 (16.4%)	246 (82.3%)	295
**Total**		299	299	598

*The 2 × 2 contingency table given above shows the probability of classifying the RBCs in malaria as normocytic or eryptotic based on the “Euler number” feature. The sensitivity and specificity of the tool were 83.6 and 82.3%, respectively, the PPV and NPV were calculated to be 82.5 and 83.4%, respectively and the agreement for “Euler number” feature for identifying RBCs as eryptotic or not, using this tool was found to be 0.66 (κ value), with 95% CI (0.60-0.72).*

**FIGURE 6:**
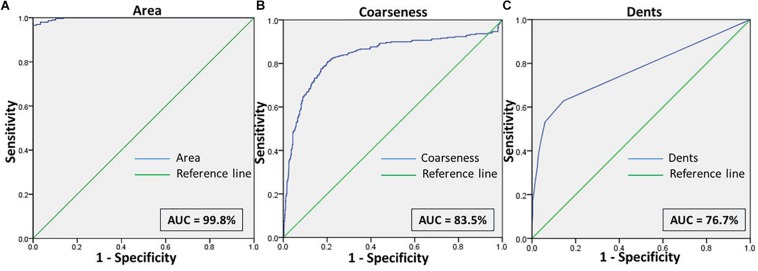
Shows the ROC curves for the features **(A)** Area, **(B)** Coarseness, and **(C)** Dents between RBCs of healthy samples in NR and malaria samples in NaCl.

**FIGURE 7:**
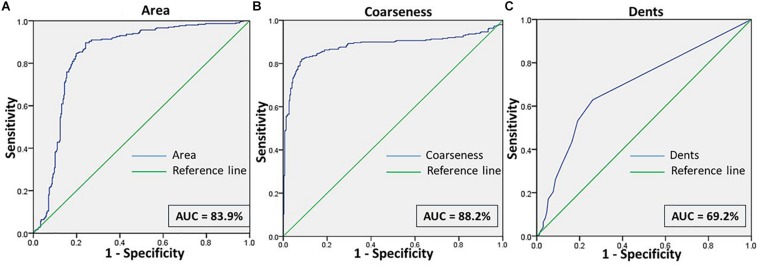
Shows the ROC curves for the features **(A)** Area, **(B)** Coarseness, and **(C)** Dents between RBCs of malaria samples in NR and NaCl.

Comparisons between RBCs of healthy samples in NR and malaria samples in NaCl too yielded very positive test characteristics for all features (Figures 3, 6 and Table 1). The sensitivity, specificity, PPV and AUC for the feature “area” were 99.7, 89.8, 89.9, and 99.8%, respectively, with very good likelihood ratio (9.77). “Coarseness” depicted high sensitivity (80.0%), specificity (99.4%), AUC (83.5%) and an excellent likelihood ratio (133.3). Likewise, “dents” too furnished fairly good set of test characteristics with high sensitivity (70.0%), specificity (72.2%) and an AUC of 76.7%. The likelihood ratio was calculated to be 4.86. Figure 6 presents the ROC curve for “area,” “coarseness” and “dents” and Table 2 gives the test characteristics for these three features. The “Euler number” feature also was significantly different (*p* < 0.001) between the groups, as given in Table 5.

**TABLE 5:** Distribution of eryptotic RBCs, based on “Euler number” feature between RBCs of healthy samples with unstressed and stressed RBCs of malarial samples.

**Group**	**RBCs with abnormal Euler number**	***P*-value**
1 vs. 3	41/990 (4.1%)	<0.001
	53/299 (17.7%)	
1 vs. 4	41/990 (4.1%)	<0.001
	250/299 (83.6%)	

*In the probability distribution table given above, comparisons between RBCs of healthy samples in NR (Group 1) was made with RBCs of malarial samples in NR (Group 3) and hyperosmotic NaCl (Group 4), with respect to the presence/absence of the central halo, computed by the “Euler number” feature. Test results displayed a significantly higher number of eryptotic RBCs in Groups 3 (*p* < 0.0001) and an even greater number of eryptotic RBCs in Group 4 (*p* < 0.0001) when compared with Group 1.*

## Discussion

An important breakthrough in biology is the knowledge that the RBC, although organelle-less, can undergo a programmed self-destruction process called eryptosis (Bosman et al., 2005). The reports claimed in this paper confirms the observations in previous studies that hyperosmotic shock-induced-eryptosis produces structural variations such as cell shrinkage and cell vesiculations. The mechanisms by which hyperosmotic stress induces these morphological modifications are summarized as follows (details abstracted from articles by Lang et al.). Hyperosmotic shock causes an increased influx of Ca^2+^ across the erythrocyte cell membrane through non-selective cation channels (Lang et al., 2003). Hyperosmotic shock can also stimulate the release of prostaglandin E2, which would, in turn, further augment Ca^2+^ entry (Lang P.A. et al., 2005). Increase in cytosolic Ca^2+^ concentration stimulates the Gardos channels, resulting in K^+^ efflux, hyperpolarization of the cell membrane and Cl^–^ exit, paralleled with K^+^ efflux. Cellular loss of KCl is followed by the exit of osmotically obliged water, leading to cell shrinkage (Lang K.S. et al., 2005). Increased cytosolic Ca^2+^ stimulates membrane vesiculation and cell membrane scrambling (trans-bilayer movement of membrane phospholipids), leading to phosphatidylserine exposure at the cell surface (Williamson et al., 1992). Ca^2+^ also stimulates degradation of the RBC cytoskeleton, facilitating cell blebbing, by activating calpain (Pant et al., 1983).

We made an attempt to apply image analysis, to decipher multiple morphological variations in RBCs, when they were stressed hyperosmotically. The RBC shrinkage (area), formation of vesiculations (dents and coarseness) and disappearance of the central halo (Euler number) were the striking observational features (Figures 1, 2) that were picked up by the designed computer program. These observations were not new in any way and its already been established that cell shrinkage and membrane blebbings are hallmark characteristics of eryptosis (Lang et al., 2004). But the novelty of this study is that we considered quantifying the extent of these morphological changes. With the customized programs that had been written in MATLAB to detect RBCs and further compute and quantify the morphological features from them, we obtained clear and significant results (Figure 1 and Tables 1, 3) for the categorization of normocytic and eryptotic RBCs, based on “area,” “dents,” “coarseness” and Euler number. Using this approach, we analyzed a total of 1,912 RBCs, of which 70% (1,338) of randomly selected RBCs were included in the training set for the generation of algorithms and 30% (574) of the total RBCs were included in the testing set. The ROC curve results indicated an acceptable AUC with good sensitivity, specificity and PPV values for each of the morphometric parameters “area,” “dents” and “coarseness” (Figure 4 and Table 2) and so did the contingency Table 3 for “Euler number.”

Validation of the method was done on blood samples from patients with malaria (*n* = 17). For this, we have analyzed 598 RBCs, from confirmed malarial blood samples. Previous studies have verified the presence of eryptosis in malarial infection. The results of our study report that the algorithms that we have generated detected a significant number of eryptotic RBCs in malarial samples, through the computation of “area,” “coarseness,” “dents,” and “Euler number” (Figure 3 and Tables 1, 4, 5). This confirms that eryptosis in malaria can be detected and measured by this approach. Hyperosmotic stress of RBCs from malarial samples resulted in augmented eryptotic features, suggesting that an eryptotic infection such as malaria, when acted upon by another eryptotic stressor such as hyperosmotic shock, induces amplified eryptosis. The ROC curves generated for the features “area,” “coarseness,” and “dents” yielded good and acceptable test characteristics (Figures 5–7 and Table 2). Results of the “Euler number” feature (Table 4) show that hyperosmotic stress of RBCs of malarial samples resulted in the significant loss of the central halo. When comparisons between the “Euler number” feature among RBCs of healthy samples were made with RBCs of malarial samples, it was found that significant number of RBCs appeared to have lost their central halo in malaria, whether unstressed or stressed with hyperosmotic shock (Table 5).

Cell imaging in medical research have progressed a considerable level and is undoubtedly most accurate by several advanced technologies, by which data obtained are highly precise and reliable. However, the downsides of these gold-standard tools are that they are expensive, not always quantitative and necessitate extensive efforts for appropriate sample processing and preparation (Inkson, 2016). These conventional methods, because of its cumbersome procedures, consumes considerable lengths of the researcher’s time and may require the assistance of multiple laboratory personnel. In that line, the method we have proposed here is simple and inexpensive, as it does not require any sample-fixing procedures or elaborate laboratory set-ups, is automated and thus multiple samples can be assessed in a concise time and does not necessitate much manual labor. Existing software models provide means of detecting, classifying and counting normal and abnormal RBCs. Recently, attempts have been made to produce automated tools for detection of red cell deformities in peripheral smear, especially in detection of malaria (Oliveira et al., 2017) and certain hemoglobinopathies such as sickle cell anemia (Xu et al., 2017) and G6PD deficiency hemolytic anemia (Bancone et al., 2018). However, there exists a dearth in the effectual computational algorithm for morphometric quantification of abnormalities, such as seen in eryptosis. Automatic image analysis can definitely detect variations not discernible to our vision, and there is a need for a novel and robust software model that is cost-effective, convenient and capable in assessing eryptosis. There had been similar attempts to compute morphometric characteristics and its variations in RBCs earlier, such as for classification of normocytic and abnormal RBCs in healthy and anemic patient samples using “chain-code analysis,” utilizing the very same parameters we had chosen in the current study, viz, cell size, number of spicules and the presence or absence of the central halo (Bacus and Weens, 1977). With more modern technology now available, the results in our study promises and produces a better outcome than that was available in earlier studies.

## Conclusion

Measuring RBC size and gauging the degree of its structural abnormality by manual microscopic inspection, although crucial, has always been a complex and tedious job for a hematologist, often, unfortunately, resulting in inaccurate results (Sharma, 2017). Also, it requires the review of multiple images per sample, which is quite a laborious task, taking a considerable amount of time just for analysis (Mohammed et al., 2014).

This kind of high throughput image analysis tool, based on simple software programs, can be used in a laboratory set-up for day-to-day monitoring of blood samples, blood banks and also community programs, where quick results of a large number of cases need to be handled. These algorithms could also be integrated into simple opto-digital microscopes, portable pocket microscope or wireless handheld microscope for mobile, iPod, iPhone, and iPad devices in a telemedicinal system or even hand-friendly microscopic gadgets. For hematological research, especially in laboratories with less infrastructure and facilities, this tool can give detailed information on cell morphometries. The computerized approach we report in this paper to study eryptosis demonstrates an easily adaptable means of learning structural variations of RBCs and eryptosis. The obtained experimental results validate the proposed method and demonstrate its applicability. Nevertheless, we have assessed the feasibility of this technique in malaria. This tool needs to be further validated for other stressors and pathologies on RBC morphometrics, thereby improving and expanding its features. In conclusion, this abstract model represents a primary step toward expanding our understanding of eryptosis and can pave way to learn more on the behavior and shift of RBC structure to different milieus and pathology.

## Data Availability Statement

The datasets generated and analyzed during this study are available from the corresponding author on reasonable request.

## Ethics Statement

The studies involving human participants were reviewed and approved by Kasturba Medical College and Kasturba Hospital Institutional Ethics Committee, Reg no. ECR/146/Inst/KA/2013/RR-16. The participants provided their written informed consent to participate in this study.

## Author Contributions

SJ and KP conceived the study, designed and performed the experiments, interpreted results and wrote the manuscript. PR and RR contributed to the conception and design of the project and interpretation of results. AK carried out the statistical analysis, interpretation of results, and wrote the manuscript. RH performed the software development, analysis, and delivered the data. PB assisted with the design of the experiments and interpretation of results.

## Conflict of Interest

The authors declare that the research was conducted in the absence of any commercial or financial relationships that could be construed as a potential conflict of interest.
